# Supplementary material for: Greatwall kinase regulates acute myeloid leukaemia cell division through a non-canonical mechanism
Source: EMBO Rep. 2026 Sep 30;27(21):6464–88. doi: 10.1038/s44319-026-00955-6 (PMC13743553; doi:10.1038/s44319-026-00955-6)
Supplement: Supplementary file 1 [file 44319_2026_955_MOESM12_ESM.pdf]

Expanded View Figures

Figure EV1. Effect of Gwl inhibition on AML cell proliferation.

(**A**) Relative cell number of AML cells lines treated with different concentrations of Gwl inhibitor (c604) for 72 h. Cell number is expressed as relative to DMSO treated cells. Values represent mean ± SD of three independent biological replicates. (**B**) Bar plots showing the response to c604 calculated as Area Above the Curve (AAC) from the data presented in (**A**). Bar plots show the mean ± SD ($n = 3$ independent biological replicates). (**C**) Gwl genetic dependency of cancer cell lines grouped by primary disease from RNAi Achilles Dataset ($n = 458$ cell lines). Statistical significance was calculated by unpaired *t* test between AML versus the rest of the cancer types (**P* < 0.05, ***P* < 0.01 and ****P* < 0.001). (**D**) Abundance of named proteins across the 14 AML cell lines tested and normalised signal intensity for the phosphorylation of ENSA/ARPP19 at Ser62 and Ser67 obtained from (Gerdes et al, 2021) ($n = 3$ independent biological replicates from the original dataset). (**E**) Distributions of cell cycle groups (2 N, S, 4 N, 4 N+ and <2 N) in AML cells treated with c604 (0.25 µM) for 24 and 48 h of three independent biological replicates. Statistical significance was calculated by unpaired *t* test between treated cells versus non-treated (DMSO) groups (**P* < 0.05, ***P* < 0.01 and ****P* < 0.001). (**F**) Sequence motifs of significant decreased phosphopeptides in mitotic HeLa cells treated with c604. (**G**) Box plots showing the relative protein abundance of Gwl (MASTL), ENSA, ARPP19, B55α (PPP2R2A), CIP2A, SET and PP2A (PPP2CA) in AML cases and healthy donors (HD). Phosphorylation levels (ppIndex) for ENSA/ARPP19 phosphorylation are also shown. Dots represent individual AML patients or healthy donors (HD). Data from (Casado et al, 2023). Unpaired *t* test was used to calculate the differences between HD and AML groups (**P* < 0.05, ***P* < 0.01 and ****P* < 0.005). (PPP2R2A $P = 0.0021$; ARPP19 $P = 4.6e\text{-}07$; CIP2A $P = 0.93$; ENSA $P = 1.3e\text{-}15$; PPP2CA $P = 0.033$; SET $P = 5.3e\text{-}07$; ARPP19(S62) $P = 3.2e\text{-}09$). Box plots in (**C**, **D**) show the median (centre), 25th and 75th percentiles (lower and upper bounds of the box), and whiskers extending to the minimum and maximum values within 1.5× the interquartile range. Source data are available online for this figure.

© The Author(s)

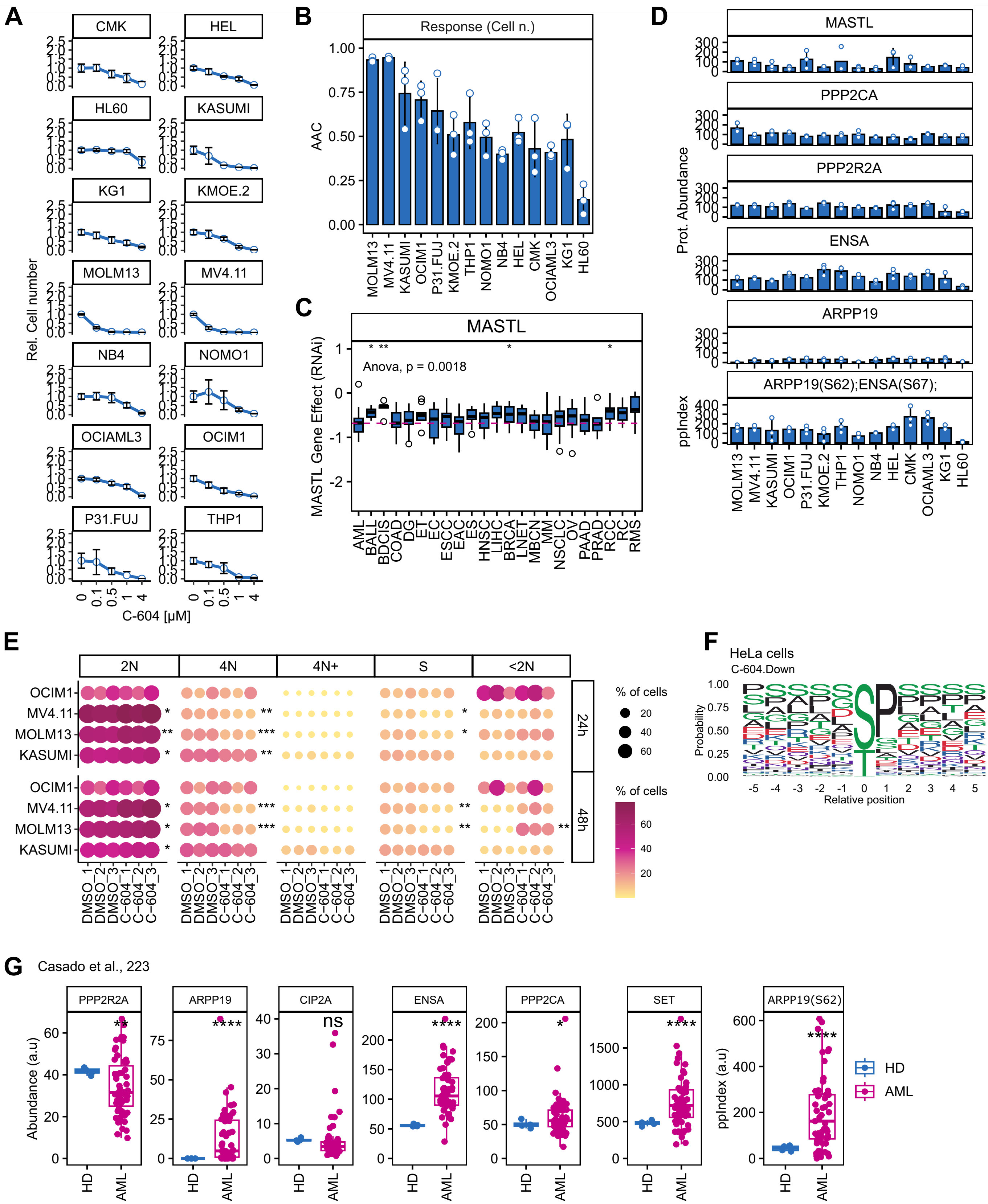
A
CMK
HEL
HL60
KASUMI
KG1
KMOE.2
MOLM13
MV4.11
NB4
NOMO1
OCIAML3
OCIM1
P31.FUJ
THP1
Rel. Cell number
C-604 [μM]
B
Response (Cell n.)
AAC
C
MASTL
Anova, p = 0.0018
MASTL Gene Effect (RNAi)
D
MASTL
PPP2CA
PPP2R2A
ENSA
ARPP19
ARPP19(S62);ENSA(S67);
Prot. Abundance
ppIndex
E
2N
4N
4N+
S
<2N
24h
48h
% of cells
F
HeLa cells
C-604.Down
Probability
Relative position
G
Casado et al., 223
PPP2R2A
ARPP19
CIP2A
ENSA
PPP2CA
SET
ARPP19(S62)
Abundance (a.u)
ppIndex (a.u)
HD
AML

 © The Author(s)

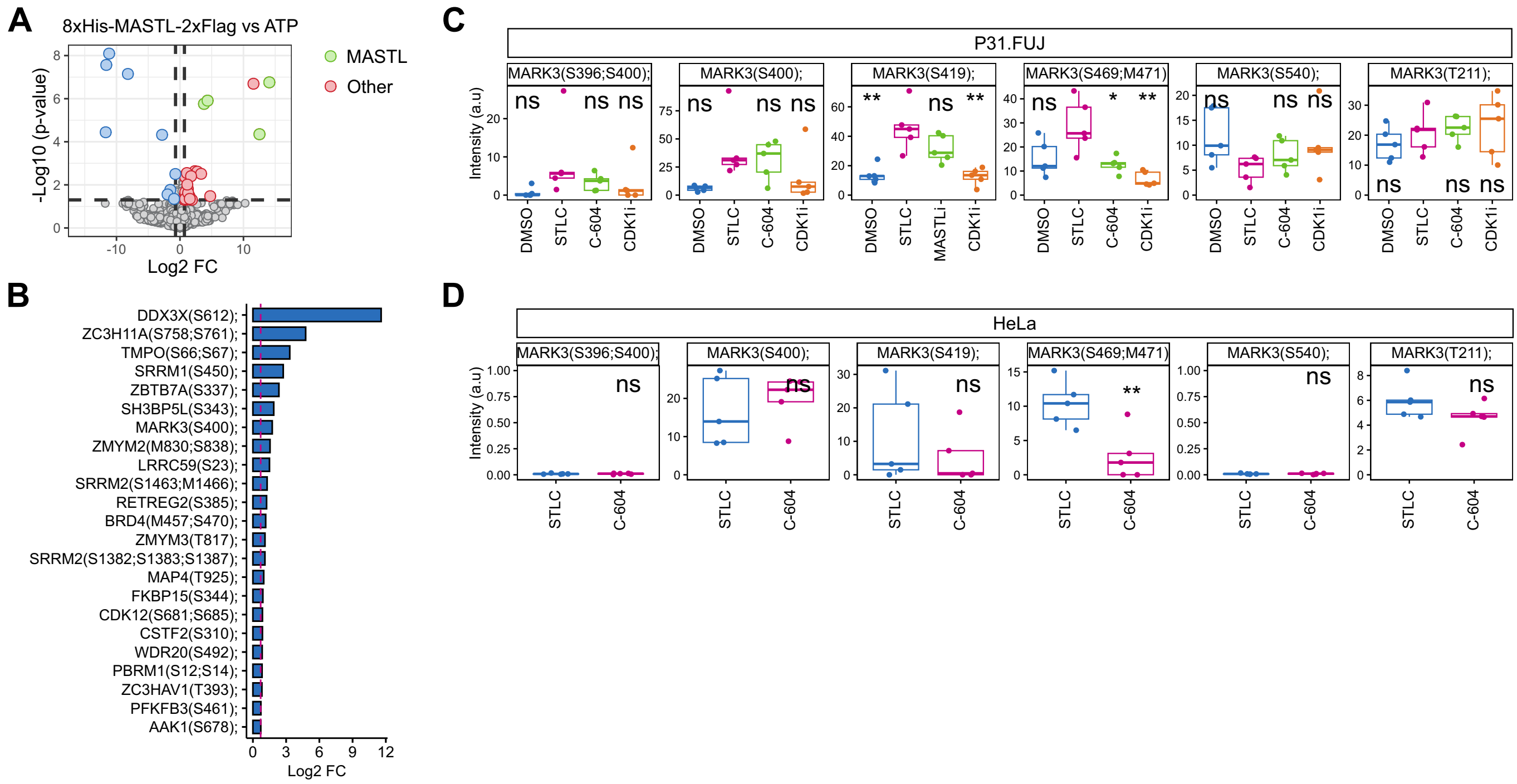

Figure EV2. MARK3 phosphorylation by Gwl.

(**A**) Volcano plot shows phosphopeptides identified by LC-MS/MS in the kinase assay on fixed P31/Fuj cells. *X* axis represents the FC (Log2 FC) of cells incubated with 8xHis-Gwl-2xFLAG versus cells incubated only with ATP ($n = 3$ independent biological replicates). Blue and red colours indicate significant ($P < 0.05$) phosphopeptides with decreased ($FC < -0.7$) and increased ($FC > 0.7$) phosphorylation compared to ATP only, respectively. Green dots show Gwl phosphopeptides. (**B**) Significant phosphopeptides identified in the kinase assay from A ranked by FC. Dashed pink line indicates the Log2 FC threshold of 0.7. Statistical differences were assessed using LIMMA. (**C**) Box plot shows the normalised signal intensity for MARK3 phosphopeptides identified in P31/Fuj cells by LC-MS/MS ($n = 5$ independent biological replicates). "*MARK3(S419);*" DMSO $P = 0.0084$, c604 $P = 0.133$, CDK1i $P = 0.0073$; "*MARK3(S469;M471)*" DMSO $P = 0.0556$, c604 $P = 0.0266$, CDK1i $P = 0.0089$. (**D**) Box plot shows the normalised signal intensity for MARK3 phosphopeptides identified in HeLa cells by LC-MS/MS. ($n = 5$ independent biological replicates). Unpaired *t* test was used to calculate the differences between the different treatments versus STLC treatment (mitotic) (*$P < 0.05$ and **$P < 0.01$). "*MARK3(S469;M471)*" $P = 0.0087$. Box plots in (**C**, **D**) show the median (centre), 25th and 75th percentiles (lower and upper bounds of the box), and whiskers extending to the minimum and maximum values within 1.5× the interquartile range. Source data are available online for this figure.

© The Author(s)
