# Supplementary material for: Greatwall kinase regulates acute myeloid leukaemia cell division through a non-canonical mechanism
Source: EMBO Rep. 2026 Sep 30;27(21):6464–88. doi: 10.1038/s44319-026-00955-6 (PMC13743553; doi:10.1038/s44319-026-00955-6)
Supplement: Supplementary file 2 [file 44319_2026_955_MOESM1_ESM.pdf]

EMBO reports

Greatwall Kinase regulates Acute Myeloid Leukaemia Cell Divi- sion through a Non-Canonical Mechanism

Sandra Martin-Guerrero, Tommy Shields, Pedro Casado, Róbert Zach, Vinothini Rajeeve, Nadia Afroz-Nishat, Irbaz Badshah, Megan Meredith, William Foster, Helfrid Hochegger, and Pedro Cutillas

Corresponding author(s): Pedro Cutillas (prcutillas@ipb.csic.es) , Helfrid Hochegger (hh65@sussex.ac.uk), Sandra Martin-Guerrero (sandrammg92@ipb.csic.es)

Review Timeline:

Submission Date:	20th Apr 26
Editorial Decision:	2nd Jul 26
Revision Received:	10th Aug 26
Editorial Decision:	9th Sep 26
Revision Received:	15th Sep 26
Accepted:	18th Sep 26

Editor: Kurt Weir / Achim Breiling

Transaction Report:

(Note: With the exception of the correction of typographical or spelling errors that could be a source of ambiguity, letters and reports are not edited. Depending on transfer agreements, referee reports obtained elsewhere may or may not be included in this compilation. Referee reports are anonymous unless the Referee chooses to sign their reports.)

Dear Prof. Hochegger

Thank you for the submission of your research manuscript to our journal. We have now received the full set of referee reports that is copied below.

As you will see, the referees acknowledge that the findings are interesting and that the conclusions are overall supported by the data presented but they also raise a number of concerns and have suggestions how to further strengthen the data, particularly the inclusion of specific experiments to directly support your proposed mechanism. EMBO Reports will not require the inclusion of xenograft experiments.

Given these constructive comments, we would like to invite you to revise your manuscript with the understanding that the referee concerns (as detailed above and in their reports) must be fully addressed and their suggestions taken on board. Please address all referee concerns in a complete point-by-point response. Acceptance of the manuscript will depend on a positive outcome of a second round of review. It is EMBO Reports policy to allow a single round of revision only and acceptance or rejection of the manuscript will therefore depend on the completeness of your responses included in the next, final version of the manuscript.

We realize that it is difficult to revise to a specific deadline. In the interest of protecting the conceptual advance provided by the work, we recommend a revision within 3 months (October 2nd). Please discuss the revision progress ahead of this time with the editor if you require more time to complete the revisions.

I am also happy to discuss the revision further via e-mail or a video call, if you wish.

You can either publish the study as a short report or as a full article. For short reports, the revised manuscript should not exceed 27,000 characters (including spaces but excluding materials & methods and references) and 5 main plus 5 expanded view figures. The results and discussion sections must further be combined, which will help to shorten the manuscript text by eliminating some redundancy that is inevitable when discussing the same experiments twice. For a normal article there are no length limitations, but it should have more than 5 main figures and the results and discussion sections must be separate. In both cases, the entire materials and methods must be included in the main manuscript file.

================
IMPORTANT NOTE:

We perform an initial quality control of all revised manuscripts before re-review. Your manuscript will FAIL this control and the handling will be delayed IN CASE the following APPLIES:

1) A data availability section providing access to data deposited in public databases is missing. If you have not deposited any data, please add a sentence to the data availability section that explains that.

2) Your manuscript contains statistics and error bars based on n=2. Please use scatter blots in these cases. No statistics should be calculated if n=2.

When submitting your revised manuscript, please carefully review the instructions that follow below. Failure to include requested items will delay the evaluation of your revision.

================

When submitting your revised manuscript, we will require:

1) a .docx formatted version of the manuscript text (including legends for main figures, EV figures and tables). Please make sure that the changes are highlighted to be clearly visible.

2) individual production quality figure files as .eps, .tif, .jpg (one file per figure).
Please download our Figure Preparation Guidelines (figure preparation pdf) from our Author Guidelines pages https://www.embopress.org/page/journal/14693178/authorguide for more info on how to prepare your figures.

3) a .docx formatted letter INCLUDING the reviewers' reports and your detailed point-by-point responses to their comments. As part of the EMBO Press transparent editorial process, the point-by-point response is part of the Review Process File (RPF), which will be published alongside your paper.

4) a complete author checklist, which you can download from our author guidelines

(<https://www.embopress.org/page/journal/14693178/authorguide>). Please insert information in the checklist that is also reflected in the manuscript. The completed author checklist will also be part of the RPF.

5) Please note that all corresponding authors are required to supply an ORCID ID for their name upon submission of a revised manuscript (<https://orcid.org/>). Please find instructions on how to link your ORCID ID to your account in our manuscript tracking system in our Author guidelines (<https://www.embopress.org/page/journal/14693178/authorguide#authorshipguidelines>)

6) We replaced Supplementary Information with Expanded View (EV) Figures and Tables that are collapsible/expandable online. A maximum of 5 EV Figures can be typeset. EV Figures should be cited as 'Figure EV1, Figure EV2" etc... in the text and their respective legends should be included in the main text after the legends of regular figures.

- For the figures that you do NOT wish to display as Expanded View figures, they should be bundled together with their legends in a single PDF file called *Appendix*, which should start with a short Table of Content. Appendix figures should be referred to in the main text as: "Appendix Figure S1, Appendix Figure S2" etc. See detailed instructions regarding expanded view here: <https://www.embopress.org/page/journal/14693178/authorguide#expandedview>

- Additional Tables/Datasets should be labeled and referred to as Table EV1, Dataset EV1, etc. Legends have to be provided in a separate tab in case of .xls files. Alternatively, the legend can be supplied as a separate text file (README) and zipped together with the Table/Dataset file.

7) Please include a dedicated "Data Availability" section at the end of the Methods (suggested wording: "The [structural coordinates | microarray | mass spectrometry] data from this publication have been deposited to the [name of the database] database [URL] and assigned the identifier [accession | permalink | hashtag]."). Should this not apply, this should still be stated as "This study includes no data deposited in external repositories."

8) At EMBO Press we ask authors to provide source data for the main manuscript figures. You will receive a separate email with instructions for providing source data with your revised manuscript, including how to upload and organize the files.

Additional information on source data and instruction on how to label the files are available <https://www.embopress.org/page/journal/14693178/authorguide#sourcedata>

9) The journal requires a statement specifying whether or not authors have competing interests (defined as all potential or actual interests that could be perceived to influence the presentation or interpretation of an article). In case of competing interests, this must be specified in your disclosure statement. Further information: https://www.embopress.org/competing-interests

10) Figure legends and data quantification:
The following points must be specified in each figure legend:

- the name of the statistical test used to generate error bars and P values,
- the EXACT p-values,
- the number (n) of independent experiments (please specify technical or biological replicates) underlying each data point,
- the nature of the bars and error bars (s.d., s.e.m.)

- If the data are obtained from n {less than or equal to} 5, show the individual data points in addition to the SD or SEM.
- If the data are obtained from n {less than or equal to} 2, use scatter blots showing the individual data points.

Discussion of statistical methodology can be reported in the materials and methods section, but figure legends should contain a basic description of n, P and the test applied.

See also the guidelines for figure legend preparation:
https://www.embopress.org/page/journal/14693178/authorguide#figureformat

- Please also include scale bars in all microscopy images and define their size in the legend, not the image.

11) Our journal encourages inclusion of *data citations in the reference list* to directly cite datasets that were re-used and obtained from public databases. Data citations in the article text are distinct from normal bibliographical citations and should directly link to the database records from which the data can be accessed. In the main text, data citations are formatted as follows: "Data ref: Smith et al, 2001" or "Data ref: NCBI Sequence Read Archive PRJNA342805, 2017". In the Reference list, data citations must be labeled with "[DATASET]". A data reference must provide the database name, accession number/identifiers and a resolvable link to the landing page from which the data can be accessed at the end of the reference. Further instructions are available at <https://www.embopress.org/page/journal/14693178/authorguide#referencesformat>.

12) All Materials and Methods need to be described in the main text using our 'Structured Methods' format. According to this

format, the Methods section includes a Reagents and Tools Table (listing key reagents, experimental models, software and relevant equipment and including their sources and relevant identifiers) followed by a Methods and Protocols section describing the methods, ideally using a step-by-step protocol format. The aim is to facilitate adoption of the methodologies across labs. Please download and fill our Reagents and Tools Table template (.docx), which you can find in our author guidelines: https://www.embopress.org/page/journal/14693178/authorguide#structuredmethods.
When submitting your revised manuscript, please do not include the Reagents and Tools Table in the Methods section of the manuscript but upload it as a separate file choosing the file type "Reagent Table".
An example of a Method paper with Structured Methods can be found here: https://www.embopress.org/doi/10.15252/msb.20178071.

13) As part of the EMBO publication's Transparent Editorial Process, EMBO Reports publishes online a Review Process File to accompany accepted manuscripts. This File will be published in conjunction with your paper and will include the referee reports, your point-by-point response and all pertinent correspondence relating to the manuscript.

You are able to opt out of this by letting the editorial office know (emboreports@embo.org). If you do opt out, the Review Process File link will point to the following statement: "No Review Process File is available with this article, as the authors have chosen not to make the review process public in this case."

We would also welcome the submission of cover suggestions, or motifs to be used by our Graphics Illustrator in designing a cover.

I look forward to seeing a revised form of your manuscript when it is ready and please let me know if you have questions or comments regarding the revision.

Yours sincerely,

Kurt Weir
Editor
EMBO Reports

Referee #1:

Review EMBO reports

This paper investigates the role of the Gwl/MASTL-ENSA/ARPP19 pathway in AML cells. The rational for conducting the work is that Gwl/MASTL has been described as a vulnerability in AML and thus there is a potential therapeutic potential for Gwl/MASTL inhibitors.
The authors analyse a panel of AML cells and test the effect of a recently described Gwl/MASTL inhibitor named c604. 13 out of 14 cell lines are sensitive and sensitivity correlates with the expression levels of PP2A catalytic subunit. Further cell cycle analysis following c604 treatment shows a range of phenotypic effects in the different AML cell lines and differential impacts on the phosphoproteome.
The authors show that ENSA phosphorylation in AML cells is not under the control of Gwl/MASTL and that binding of ENSA to PP2A-B55 is not affected by c604 treatment. Phosphoproteomic analysis of c604 treated cells points the authors towards proteins involved in cytokinesis as substrates of Gwl/MASTL in AML cells.
Overall, the work is interesting and nicely conducted but is rather descriptive. I am not an AML expert so cannot evaluate the impact on the field and whether the AML parts of the work are conducted properly.
I have the following main points that I think would strengthen the paper and make it more interesting:

1) There are additional kinases in the MAST kinase subfamily (MAST1-4) with MAST3 reported to phosphorylate ARPP19 in neurons. It would be interesting to test with RNAi or gRNAs if one of these kinases are responsible for phosphorylating ENSA.
2) The authors mention that sensitivity to c604 correlates with PP2A catalytic subunit expression but do not pursue this further. Is sensitivity to c604 affected by partial RNAi of PPP2CA? The results do suggest some links between MASTL and PP2A in AML cells.
3) Is the association of ENSA and PP2A-B55 in AML cells dependent on phosphorylation (relates to point 1)). One could lambda phosphatase treat the IPs to see.
4) The overlap between c604 targets and shMASTL targets (figure 4M) is a little concerning. Could it be that the c604 has some off target effects in AML cells that is not seen in other cell types? Could this be tested by treating shMASTL cells with c604? Some further evidence of c604 specificity in AML cells would be great.
5) It would be interesting to explore the functional role of MARK3 phosphorylation for AML cell viability.

Referee #2:

Overall, this is a strong and interesting manuscript that addresses a question regarding the role of Greatwall kinase (Gwl) in AML biology. The authors combine pharmacological inhibition, inducible genetic perturbation, phosphoproteomics, and functional assays to connect cellular phenotypes with molecular pathways. In particular, the work provides evidence that Gwl regulates AML cell proliferation and cytokinesis through mechanisms that appear distinct from the canonical ENSA-PP2A-B55 axis described in other systems. The study is technically well executed and contains several potentially important observations regarding context-dependent Gwl signalling in AML, including differences associated with FLT3-ITD status.

At the same time, the manuscript currently presents several mechanistically distinct models without fully integrating them into a unified framework. In FLT3-ITD AML models, Gwl inhibition appears associated with altered STAT5 phosphorylation and cell death, whereas in non-FLT3-ITD AML cells the dominant phenotype is mitotic/cytokinetic failure associated with altered phosphorylation of MARK3 and cytoskeletal regulators. In contrast, HeLa cells appear to retain dependence on the canonical ENSA-PP2A-B55 pathway. These observations are highly interesting and potentially represent the most conceptually important aspect of the manuscript, but at present they remain largely descriptive and correlative.

A key outstanding question is therefore what determines pathway selection downstream of Gwl in different cellular contexts. The manuscript would be substantially strengthened by experiments directly testing whether FLT3-ITD signalling is sufficient to alter Gwl-dependent cellular outcomes. For example, expression of FLT3-ITD in FLT3-WT AML cells, or systematic modulation of FLT3/STAT5 signalling across models, combined with assessment of STAT5 phosphorylation, apoptosis, cytokinesis defects, and MARK3 phosphorylation, could help establish whether oncogenic FLT3 signalling rewires Gwl dependency. While FLT3-ITD expression alone may not fully recapitulate AML signalling context, even a partial phenotypic shift would considerably strengthen the mechanistic model proposed by the authors.

Related to this, the manuscript raises but does not fully address the important question of why the canonical ENSA-PP2A-B55 pathway appears dispensable in AML cells. At present, the comparison with "solid tumours" relies primarily on HeLa cells, which may not sufficiently support broader conclusions regarding tumour-type-specific Gwl signalling. The authors should therefore either expand this analysis or temper the scope of these conclusions. At minimum, the Discussion would benefit from a more explicit consideration of potential explanations for this apparent rewiring, including lineage-specific phosphatase regulation, compensatory phosphatase networks, altered substrate accessibility, or effects of oncogenic FLT3 signalling on mitotic phosphoregulation.

Similarly, the statement:
"These findings indicate that MARK3 regulation may differ across tumour types and suggest that Gwl controls MARK3 phosphorylation and activity in AML."
currently appears somewhat overgeneralized given the heterogeneity reported between AML models themselves. The conclusions regarding tumour-type-specific MARK3 regulation should therefore be moderated accordingly.

Another important point concerns interpretation of the phosphoproteomic data. Some of the phosphorylation changes observed following Gwl inhibition or depletion may reflect secondary consequences of mitotic/cytokinetic disruption rather than direct Gwl substrates. Since MARK3 is proposed as a direct Gwl substrate, it would be important to clearly distinguish direct versus indirect effects. Depending on what data are already available, this could include stronger biochemical validation (e.g. in vitro kinase assays, phosphosite validation, rescue approaches with phospho-mutants, or temporal analyses prior to overt mitotic collapse).

Finally, an important technical consideration concerns the inducible shRNA depletion strategy used throughout the study. Inducible knockdown approaches inherently depend on endogenous protein turnover kinetics and may therefore incompletely capture acute requirements for Gwl function. This limitation may also contribute to discrepancies between genetic depletion and pharmacological inhibition phenotypes. While this may not necessarily require additional experimentation, the manuscript would benefit from explicit discussion of this limitation. Alternatively, more acute degradation strategies (e.g. degron-based approaches) could provide additional mechanistic clarity.

Minor comments:

Several numerical expressions in the Materials and Methods section appear inconsistently formatted (e.g. "0.5 × 106" instead of superscript notation), which occasionally affects readability.

Referee #3:

Summary:
This study identifies Greatwall kinase as a critical regulator of AML cell growth and division through a previously unrecognized mechanism. Rather than acting through its canonical ENSA-PP2A-B55 signaling pathway, Greatwall supports proper cytokinesis by controlling the phosphorylation of proteins involved in cytoskeletal dynamics. The discovery of MARK3 as a direct Greatwall

target highlights a distinct signaling network in leukemia cells and suggests that targeting Greatwall may represent a novel therapeutic strategy for AML.

This study provides intriguing evidence for a novel role of Greatwall kinase in AML cell-cycle regulation. However, additional mechanistic and functional experiments are needed to further validate the proposed pathway and strengthen the conclusions regarding Greatwall's role in cytokinesis and MARK3 regulation.

Comments:
1. Although the authors tested 14 AML cell lines, most mechanistic experiments (phosphoproteomics, knockdown studies, MARK3 analyses, ENSA studies) were performed primarily in P31/Fuj cells, with limited validation in a few additional lines. AML cell lines often have chromosomal instability and abnormal mitotic regulation, making it difficult to determine whether the observed cytokinesis defects are specific consequences of Greatwall inhibition or reflect pre-existing vulnerabilities.

2. The central mechanistic claim of the study is that Greatwall regulates cytokinesis through MARK3; however, the evidence presented remains largely correlative rather than causative. The authors show that Greatwall can phosphorylate MARK3 in vitro, that treatment with c604 reduces MARK3 phosphorylation, and that phosphorylation of downstream MARK3 substrates is altered under these conditions. While these findings are consistent with a potential regulatory relationship, they do not establish a direct functional requirement for MARK3 in mediating Greatwall-dependent cytokinesis.

3. Key experiments that are currently missing include functional validation of MARK3 through loss-of-function approaches such as knockdown or knockout to test whether it is necessary for the observed cytokinesis phenotype. In addition, rescue experiments using phosphomimetic MARK3 mutants would be required to determine whether restoring MARK3 activity can overcome the effects of Greatwall inhibition. Phenocopy studies are also needed to assess whether depletion of MARK3 reproduces the cytokinesis defects seen upon Greatwall inhibition. Overall, without these genetic and rescue-based experiments, MARK3 cannot be established as a validated downstream mediator, and it should instead be regarded as a putative effector rather than a confirmed mechanistic link between Greatwall and cytokinesis.

4. This is probably the biggest weakness. The authors demonstrate reduced proliferation and viability in cell lines and primary AML samples, but they never test whether Greatwall inhibition suppresses leukemia growth in animal models. There are no xenograft studies, leukemia burden measurements, or survival analyses. Therefore, the therapeutic relevance of targeting Greatwall in AML remains speculative.

5. The study lacks key genetic rescue experiments that are essential for establishing causality in the proposed pathway. Specifically, it does not include re-expression of shRNA-resistant Greatwall to confirm that the observed phenotypes are specifically due to Greatwall depletion. In addition, expression of constitutively active MARK3 would be important to determine whether downstream signaling can be restored independently of upstream inhibition. A critical missing element is also the demonstration that cytokinesis defects can be rescued upon restoration of either Greatwall or MARK3 activity. Without these rescue experiments, it remains difficult to rule out indirect or off-target effects as the underlying cause of the observed phenotypes.

Cross-comment session
Referee 2
I agree with the comments of the other reviewers. The main area for improvement is the transition from correlative observations to a more mechanistic understanding of the reported phenomena. The suggested degron-based and animal experiments would significantly strengthen the study and provide important validation of the proposed model. Nevertheless, these experiments would likely require substantial additional work and, in my opinion, should be considered a strong addition rather than a strict requirement.

Reviewer 1

This paper investigates the role of the Gwl/MASTL-ENSA/ARPP19 pathway in AML cells. The rational for conducting the work is that Gwl/MASTL has been described as a vulnerability in AML and thus there is a potential therapeutic potential for Gwl/MASTL inhibitors.
The authors analyse a panel of AML cells and test the effect of a recently described Gwl/MASTL inhibitor named c604. 13 out of 14 cell lines are sensitive and sensitivity correlates with the expression levels of PP2A catalytic subunit. Further cell cycle analysis following c604 treatment shows a range of phenotypic effects in the different AML cell lines and differential impacts on the phosphoproteome.

The authors show that ENSA phosphorylation in AML cells is not under the control of Gwl/MASTL and that binding of ENSA to PP2A-B55 is not affected by c604 treatment. Phosphoproteomic analysis of c604 treated cells points the authors towards proteins involved in cytokinesis as substrates of Gwl/MASTL in AML cells. Overall, the work is interesting and nicely conducted but is rather descriptive. I am not an AML expert so cannot evaluate the impact on the field and whether the AML parts of the work are conducted properly.

Response:

We thank the Reviewer for the positive and constructive review.

I have the following main points that I think would strengthen the paper and make it more interesting:

1. *There are additional kinases in the MAST kinase subfamily (MAST1–4) with MAST3 reported to phosphorylate ARPP19 in neurons. It would be interesting to test with RNAi or gRNAs if one of these kinases are responsible for phosphorylating ENSA.*

Response:

The question of whether other MAST family members contribute to ENSA phosphorylation is a fascinating avenue for future research. However, the primary objective of this study was to characterize a Gwl-specific non-canonical pathway, which we unexpectedly found to oprate in AML cells. We have added a sentence to the Discussion acknowledging the possibile involvment of MAST kinases:

"With respect to the kinase(s) responsible for ENSA/ARPP19 phosphorylation in AML, members of the MAST kinase family, particularly MAST3, have been reported to phosphorylate ARPP16 (Musante *et al*, 2017; Andrade *et al*, 2017), a protein closely related to ARPP19. Notably, MAST kinases share structural homology with MASTL/Gwl (Andrade *et al*, 2017). Given this homology and their established role in ARPP16 phosphorylation, MAST kinases represent plausible candidate kinases for ARPP19 phosphorylation. Nevertheless, further studies are required to identify the kinase(s) responsible for ENSA/ARPP19 phosphorylation in AML cells."

2. *The authors mention that sensitivity to c604 correlates with PP2A catalytic subunit expression but do not pursue this further. Is sensitivity to c604 affected by partial RNAi of PPP2CA? The results do suggest some links between MASTL and PP2A in AML cells.*

Response:

We appreciate the reviewer's interest in the relationship between PP2A levels and sensitivity to c604. While this is an interesting direction, it is tangential to the main focus of our study, which centers on the role of Greatwall kinase in non-canonical signaling of AML cells.

Our data indicate that higher PPP2CA expression correlates with increased sensitivity to c604. We note that pharmacological PP2A inhibition with okadaic acid (OA) can partially reduce sensitivity to c604, although this must be interpreted with caution given the broad and pleiotropic effects of PP2A inhibition. For this same reason, interfering with PP2A activity is expected to produce data difficult to interpret. Thus, while mechanistically interesting, we consider that dissecting PP2A contributions through PPP2CA knockdown would require a dedicated project that falls outside the scope of the present investigation.

3. *Is the association of ENSA and PP2A-B55 in AML cells dependent on phosphorylation (relates to point 1)). One could lambda phosphatase treat the IPs to see.*

Response:

We agree that the phosphorylation-dependence of this interaction is a key concept. We have added clarification to the text to ensure this well-established principle (Hached et al., 2019) is clearly integrated into our manuscript of why our crosslinking approach was chosen.

Crosslinking, which we did as part of our experimental workflow, stabilizes the native complex prior to extraction. Once the proteins are crosslinked, lambda phosphatase treatment cannot dissociate the complex, even if this is phosphorylation-dependent. For these reasons, while conceptually interesting, the proposed experiment cannot be meaningfully applied to crosslinked complexes in our setup. To address this point we have added this sentence to the Results section:

"Immunoprecipitations were performed following the *in vivo* reversible crosslinking strategy described by (Hached *et al*, 2019) to stabilize the ENSA-B55α complex before cell lysis."

4. *The overlap between c604 targets and shMASTL targets (Figure 4M) is a little concerning. Could it be that c604 has some off-target effects in AML cells that are not seen in other cell types? Could this be tested by treating shMASTL cells with c604? Some further evidence of c604 specificity in AML cells would be great.*

Response:

We thank the Reviewer for noting this concern, but in our view, based on previous experience, we were not overtly surprised by the limited overlap between c604- and shMASTL- phosphorylation targets. Such incomplete overlap is likely to arise from several technical and biological factors rather than from AML-specific off-target effects.

First, RNAi-mediated knockdown is inherently incomplete. In our system, Greatwall depletion reached ~60% based on Western blot analysis (Fig. 4A), which is typical for shRNA approaches. Partial knockdown often results in attenuated responses compared with acute pharmacological inhibition.

Second, genetic inhibition represents a chronic perturbation (depletion was observed after 7 days after shRNA transfection), allowing time for downstream and compensatory pathways to become modulated. In contrast, pharmacological small molecule agents

(c604 in our study) produce an acute inhibition of their kinase targets (effects are observed after just 30 to 120 minutes after treatment). These mechanistic differences naturally reduce the overlap between the two datasets.

Third, the two perturbations were performed under different synchronization conditions. c604 treatment was applied to cells that had been synchronized before pharmacological inhibition, whereas shMASTL cells were synchronized after genetic depletion. This difference, which is inherent for each experimental system, is expected to influence the phosphoproteomics profiles.

Regarding specificity, we do not claim that c604 is completely selective for Greatwall kinase, as mentioned in the manuscript. In fact, very few kinase inhibitors are fully specific, and some degree of off-target activity is common. Nevertheless, the combination of genetic and pharmacological inhibition used in our study provides a consistent cause-and-effect relationship that goes beyond correlative observations. Together, these complementary approaches strongly support the conclusions presented in the manuscript. We have modified the manuscript to discuss further the nature of the reduced overlap between both approaches.

5. *It would be interesting to explore the functional role of MARK3 phosphorylation for AML cell viability.*

Response:

We thank the Reviewer for the suggestion to explore the functional role of MARK3. We agree that this is a key question. To address this, we have performed new genetic dependency mining and pharmacological inhibition assays (new Figure 8). These data demonstrate that while MARK3 is a direct Greatwall substrate, it is not the primary driver of the phenotype. We have updated the Results and Discussion to reflect this more nuanced interpretation. In detail, this analysis consists of:

a) Genetic dependency analyses. Results of mining large-scale CRISPR screens (new Figure 8) show that MARK3 is non-essential in AML or in other cancer cell lines. This pattern differs with the effect of CRISPR against Greatwall and indicates that loss of MARK3 function, on its own, is unlikely to generate a pronounced cytokinetic defect.

b) Pharmacological inhibition. Consistent with the gene dependency data, treatment of P31/Fuj cells with a novel MARK3 inhibitor (PCC0208017) does not reduce P31 cells' viability when it is used alone (new Figure 8B). Notably, combining MARK3 inhibition with c604 produces a modest synergistic decrease in cell viability (new Figure 8C), suggesting that MARK3 plays a secondary, modulatory role rather than acting as the primary driver of the cytokinetic phenotype.

Reviewer 2

Overall, this is a strong and interesting manuscript that addresses a question regarding the role of Greatwall kinase (Gwl) in AML biology. The authors combine pharmacological inhibition, inducible genetic perturbation, phosphoproteomics, and functional assays to connect cellular phenotypes with molecular pathways. In particular, the work provides evidence that Gwl regulates AML cell proliferation and cytokinesis through mechanisms that appear distinct from the canonical ENSA-PP2A-B55 axis described in other systems. The study is technically well executed and contains several potentially important observations regarding context-dependent Gwl signalling in AML, including differences associated with FLT3-ITD status.

Response: We thank the reviewer for noting the strengths and importance of the study and for the thoughtful and constructive review.

1. *At the same time, the manuscript currently presents several mechanistically distinct models without fully integrating them into a unified framework. In FLT3-ITD AML models, Gwl inhibition appears associated with altered STAT5 phosphorylation and cell death, whereas in non-FLT3-ITD AML cells the dominant phenotype is mitotic/cytokinetic failure associated with altered phosphorylation of MARK3 and cytoskeletal regulators. In contrast, HeLa cells appear to retain dependence on the canonical ENSA-PP2A-B55 pathway. These observations are highly interesting and potentially represent the most conceptually important aspect of the manuscript, but at present they remain largely descriptive and correlative.*

Response: We thank the reviewer for highlighting the conceptual importance of these observations. We would like to emphasize that the mechanistic insights presented in the manuscript are supported by both pharmacological and genetic inhibition of Gwl. The convergence of these independent approaches provides strong evidence for causality and demonstrates that the phenotypes observed extend beyond simple correlative associations.

Rather than representing unrelated models, the differences across FLT3-ITD AML, non-FLT3-ITD AML, and HeLa cells reflect context-dependent wiring of the Gwl–PP2A regulatory axis. This context-dependency was observed because, instead of focusing on a single cell line, our study evaluated different cellular models. In FLT3-ITD AML, Gwl inhibition impacts STAT5 signaling and cell viability; in non-FLT3-ITD AML, the dominant consequence is mitotic and cytokinetic failure associated with MARK3 and cytoskeletal regulators; and in HeLa cells, the canonical ENSA–PP2A-B55 pathway remains predominant.

We have expanded the discussion to more explicitly integrate these observations into a unified framework, clarifying how Gwl inhibition engages different downstream nodes depending on cellular context. We believe that this discovery of signaling plasticity of the Gwl-ENSA axis is an important new finding and a major strength of our study.

To address this point we have added this paragraph to the Discussion section:

"Our findings show that Gwl activity exhibits marked context-dependent signalling rather than exclusively functioning through the conserved downstream pathway described in HeLa cells and other cellular models (Zach et al, 2025; Burgess et al, 2010; Hegarat et al, 2014). By evaluating multiple AML cell lines and primary AML samples, we demonstrate that Gwl inhibition engages distinct signalling nodes depending on the cellular context: in FLT3-ITD mutant AML cells, it predominantly affects STAT5 signalling and leukemic cell viability; in non-FLT3-ITD mutant AML cells, it primarily disrupts mitotic progression and cytokinesis; independently of the canonical ENSA–PP2A-B55α pathway. These observations reveal an unexpected signalling plasticity of Gwl, highlighting its ability to regulate different downstream effectors in a context-dependent manner and representing an important conceptual advance arising from this study."

2. *A key outstanding question is therefore what determines pathway selection downstream of Gwl in different cellular contexts. The manuscript would be substantially strengthened by experiments directly testing whether FLT3-ITD signalling is sufficient to alter Gwl-dependent cellular outcomes. For example, expression of FLT3-ITD in FLT3-WT AML cells, or systematic modulation of FLT3/STAT5 signalling across models, combined with assessment of STAT5*

phosphorylation, apoptosis, cytokinesis defects, and MARK3 phosphorylation, could help establish whether oncogenic FLT3 signalling rewires Gwl dependency. While FLT3-ITD expression alone may not fully recapitulate AML signalling context, even a partial phenotypic shift would considerably strengthen the mechanistic model proposed by the authors.

Response: We appreciate the reviewer's insightful suggestion. Adressing the mechanisms that underly the observed rewiring of the ENSA/ARPP19 pathway is indeed an important question that our study raises. However to finalise this report on time we decided to follow up the other critical question of ENSA/ARPP19 independent roles of Gwl in non-FLT3-ITD AML. Adressing the pathway selection downstream of Gwl and dissecting how oncogenic FLT3 signalling modulates this dependency constitute a substantial research program, for which we are currently seeking funding.

3. *Related to this, the manuscript raises but does not fully address the important question of why the canonical ENSA-PP2A-B55 pathway appears dispensable in AML cells. At present, the comparison with "solid tumours" relies primarily on HeLa cells, which may not sufficiently support broader conclusions regarding tumour-type-specific Gwl signalling. The authors should therefore either expand this analysis or temper the scope of these conclusions. At minimum, the Discussion would benefit from a more explicit consideration of potential explanations for this apparent rewiring, including lineage-specific phosphatase regulation, compensatory phosphatase networks, altered substrate accessibility, or effects of oncogenic FLT3 signalling on mitotic phosphoregulation.*

Response: We appreciate the reviewer's thoughtful comments. The question of whether the canonical ENSA–PP2A-B55 pathway operates similarly across tumour types has already been addressed in our previous work, and in both the Introduction and Discussion we refer readers to Zach et al., Nature Communications (2025). In that study, we demonstrated that the ENSA–PP2A-B55 axis is fully operational and required for mitotic control in multiple solid tumour models, thereby providing a broader foundation beyond the present manuscript.

In the current study, HeLa cells are included strictly as a positive control for the canonical ENSA–PP2A-B55 pathway. Their role is to confirm that our experimental approaches robustly detect ENSA-dependent PP2A-B55 regulation when this pathway is active. They are not intended to serve as a representative model for solid tumours, but simply to demonstrate that the observed lack of response in AML cells was not due to technical issues. Instead of repeating experiments recently published, for an extensive characterization of other cancer models, in the article we refer the reader to Zach et al. 2025.

Our central conclusion is that AML cells exhibit a distinct wiring of the Gwl regulatory network relative to cells from other cancer types, which are well characterized elsewhere (Zach et al. 2025). Regarding, whether ENSA-PP2A-B55 may be dispensable in AML, we have extended the discussion and added additional analysis in the results section (Fig. EV1G) to investigate the potential role of ENSA-PP2A-B55 in AML. Although the main conclusions of the article is that Gwl regulates cell cyle independent of ENSA-PP2A-B55, we have revised the Discussion to also discuss this potential mechanism, as follows.

"PP2A inactivation is a well-established hallmark of AML, commonly driven by reduced expression of PP2A complex components (e.g. B55α) or by overexpression of

endogenous inhibitors such as SET and CIP2A (Cristobal *et al*, 2012, 2011; Arriazu *et al*, 2020, 2016). Our findings add an additional layer to this regulatory landscape by suggesting that elevated basal phosphorylation of ENSA/ARPP19 may contribute to further PP2A suppression in AML. Although the kinases responsible for Gwl-independent ENSA phosphorylation remain unidentified, the persistence of phosphorylated ENSA/ARPP19 bound to the PP2A-B55α complex points to a mechanism capable of maintaining PP2A inhibition independently of canonical Greatwall signalling. Consistent with this model, proteomic and phosphoproteomic analyses from poor-risk primary AML samples (Casado *et al*, 2023) reveal decreased levels of the regulatory subunit B55α (PPP2R2A), together with increased ENSA/ARPP19 expression and phosphorylation relative to healthy donors (Fig. EV1G). This combination—reduced B55α levels and heightened ENSA/ARPP19 inhibitory input—aligns with a scenario of constitutive PP2A dysregulation in AML. Taken together, these observations support the hypothesis that ENSA/ARPP19 phosphorylation represents an additional regulatory layer that contributes to PP2A inactivation in AML. This mechanism may cooperate with previously described alterations in PP2A complex composition and endogenous inhibitor expression, reinforcing the notion that PP2A suppression in AML is multifactorial and tightly controlled."

4. *Similarly, the statement: "These findings indicate that MARK3 regulation may differ across tumour types and suggest that Gwl controls MARK3 phosphorylation and activity in AML." currently appears somewhat overgeneralized given the heterogeneity reported between AML models themselves. The conclusions regarding tumour-type-specific MARK3 regulation should therefore be moderated accordingly.*

Response: We have revised the text and removed the statement.

5. *Another important point concerns interpretation of the phosphoproteomic data. Some of the phosphorylation changes observed following Gwl inhibition or depletion may reflect secondary consequences of mitotic/cytokinetic disruption rather than direct Gwl substrates. Since MARK3 is proposed as a direct Gwl substrate, it would be important to clearly distinguish direct versus indirect effects. Depending on what data are already available, this could include stronger biochemical validation (e.g. in vitro kinase assays, phosphosite validation, rescue approaches with phospho-mutants, or temporal analyses prior to overt mitotic collapse).*

Response: We agree that distinguishing direct from indirect phosphorylation events is essential for interpreting phosphoproteomic data. For this reason, the manuscript includes multiple layers of biochemical validation beyond inhibitor-based phosphoproteomics. Specifically, we performed in vitro kinase assays and in-cell kinase assays to verify that MARK3 is a direct Gwl substrate. These data are shown in Fig. 7. These complementary approaches were implemented to ensure that the conclusions are not solely based on global phosphoproteomic changes, which may indeed include secondary consequences of mitotic or cytokinetic disruption. We believe that the current

dataset provides robust biochemical validation supporting the identification of MARK3 as a direct Gwl substrate.

6. *Finally, an important technical consideration concerns the inducible shRNA depletion strategy used throughout the study. Inducible knockdown approaches inherently depend on endogenous protein turnover kinetics and may therefore incompletely capture acute requirements for Gwl function. This limitation may also contribute to discrepancies between genetic depletion and pharmacological inhibition phenotypes. While this may not necessarily require additional experimentation, the manuscript would benefit from explicit discussion of this limitation. Alternatively, more acute degradation strategies (e.g. degron-based approaches) could provide additional mechanistic clarity.*

Response: We thank the reviewer for this constructive suggestion. We agree that inducible shRNA-mediated depletion, by itself, has inherent limitations, as the efficiency and timing of knockdown depend on endogenous protein turnover. We however believe that the strength of our approach is that our study also used a relatively specific Gwl pharmacological inhibitor. The combination of genetic depletion and a small molecule inhibitor provides orthogonal means to investigate Gwl function.

To acknowledge this, we have now expanded the results section to explicitly address the temporal constraints of inducible knockdown strategies and to clarify how these factors may influence interpretation of the data. We also agree that more acute protein-degradation systems, such as degron-based approaches, could provide additional mechanistic resolution. These strategies represent an important avenue for future work, and we plan to incorporate them in follow-up studies.

7. *Minor comments: Several numerical expressions in the Materials and Methods section appear inconsistently formatted (e.g. "0.5 × 106" instead of superscript notation), which occasionally affects readability.*

Response: We thank the reviewer for the comment. We have revised the manuscript to unify the format.

Reviewer 3

This study identifies Greatwall kinase as a critical regulator of AML cell growth and division through a previously unrecognized mechanism. Rather than acting through its canonical ENSA-PP2A-B55 signaling pathway, Greatwall supports proper cytokinesis by controlling the phosphorylation of proteins involved in cytoskeletal dynamics. The discovery of MARK3 as a direct Greatwall target highlights a distinct signaling network in leukemia cells and suggests that targeting Greatwall may represent a novel therapeutic strategy for AML.

This study provides intriguing evidence for a novel role of Greatwall kinase in AML cell-cycle regulation. However, additional mechanistic and functional experiments are needed to further validate the proposed pathway and strengthen the conclusions regarding Greatwall's role in cytokinesis and MARK3 regulation.

Response: We thank the Reviewer for noting the novelty of our study and for the constructive advice.

1. *Although the authors tested 14 AML cell lines, most mechanistic experiments (phosphoproteomics, knockdown studies, MARK3 analyses, ENSA studies) were*

performed primarily in P31/Fuj cells, with limited validation in a few additional lines. AML cell lines often have chromosomal instability and abnormal mitotic regulation, making it difficult to determine whether the observed cytokinesis defects are specific consequences of Greatwall inhibition or reflect pre-existing vulnerabilities.

Response: We appreciate the reviewer's comment. We would like to emphasize that one of the strengths of the study is that, unlike many mechanistic investigations performed in a single immortalized model, we systematically examined a broad panel of AML systems, including 14 independent cell lines and primary blasts from eight patients. Rather than relying on a single model, we strived to have an experimental design that would allow us to map heterogeneity of Gwl-dependent phenotypes across genetically diverse AML contexts. We are sure the Reviewer will agree that the use of a variety of models (both cell lines and primary cells) goes beyond what is shown in most mechanistic papers.

Specifically, we assessed cell viability and multinucleated cell formation in all 14 AML lines, we performed immunoprecipitations (IPs) in two AML lines, and we evaluated ENSA phosphorylation in four AML lines and in eight primary samples. These readouts capture distinct aspects of the phenotypic and biochemical consequences of Gwl inhibition. The consistency of these effects across heterogeneous AML backgrounds strongly supports that the cytokinesis defects are not restricted to a single cell line with idiosyncratic mitotic vulnerabilities.

2. The central mechanistic claim of the study is that Greatwall regulates cytokinesis through MARK3; however, the evidence presented remains largely correlative rather than causative. The authors show that Greatwall can phosphorylate MARK3 in vitro, that treatment with c604 reduces MARK3 phosphorylation, and that phosphorylation of downstream MARK3 substrates is altered under these conditions. While these findings are consistent with a potential regulatory relationship, they do not establish a direct functional requirement for MARK3 in mediating Greatwall-dependent cytokinesis.

Response:

We appreciate the reviewer's comment and agree that the original data linking Greatwall to cytokinesis through MARK3 were not fully supported by the data. As noted, our biochemical assays demonstrate that MARK3 is a direct Gwl substrate, but this does not by itself establish that MARK3 is the principal mediator of the cytokinetic defects observed upon Gwl inhibition. To further clarify the mechanistic contribution of MARK3, we have now incorporated additional analyses:

a) Genetic dependency analyses. Results of mining large-scale CRISPR screens (new Figure 8) show that MARK3 is non-essential in AML or in other cancer cell lines. This pattern differs with the effect of CRISPR against Greatwall and indicates that loss of MARK3 function, on its own, is unlikely to generate a pronounced cytokinetic defect.

b) Pharmacological inhibition. Consistent with the gene dependency data, treatment of P31/Fuj cells with a novel MARK3 inhibitor (PCC0208017) does not reduce P31 cells' viability when it is used alone (new Figure 8B). Notably, combining MARK3 inhibition with c604 produces a modest synergistic decrease in cell viability (new Figure 8C), suggesting that MARK3 plays a secondary, modulatory role rather than acting as the primary driver of the cytokinetic phenotype.

These results indicate that, while MARK3 is a direct substrate, it is not the main effector of the cytokinetic defects triggered by Gwl inhibition. Instead, MARK3 likely plays a supportive or modulatory role within a broader Gwl regulated network.

We have revised the manuscript to reflect this more nuanced interpretation, based on new data, to emphasize that the central, well supported finding is that Gwl inhibition induces cytokinesis failure across diverse AML models, independent of the ENSA-B55 axis, and with MARK3 having a potential supporting role without being the main mediator of the phenotype.

3. Key experiments that are currently missing include functional validation of MARK3 through loss-of-function approaches such as knockdown or knockout to test whether it is necessary for the observed cytokinesis phenotype. In addition, rescue experiments using phosphomimetic MARK3 mutants would be required to determine whether restoring MARK3 activity can overcome the effects of Greatwall inhibition. Phenocopy studies are also needed to assess whether depletion of MARK3 reproduces the cytokinesis defects seen upon Greatwall inhibition. Overall, without these genetic and rescue-based experiments, MARK3 cannot be established as a validated downstream mediator, and it should instead be regarded as a putative effector rather than a confirmed mechanistic link between Greatwall and cytokinesis.

Response: We agree with the reviewer that full functional validation of MARK3, through knockdown/knockout, phenocopy, or phosphomimetic rescue experiments, would be required to establish MARK3 as a definitive downstream mediator of Greatwall. As mentioned in our response to the previous comment, new data in the revised paper demonstrate that MARK3 is a direct Gwl substrate, but they do not support MARK3 as the primary effector of the cytokinetic defects induced by Gwl inhibition.

For this reason, we argue that the genetic and rescue-based experiments suggested by the reviewer fall outside the scope of the present study because MARK3 is not essential in AML or most cancer cell lines (new Figure 8A), and because pharmacological inhibition of MARK3 alone does not impair viability, and only shows modest synergy when it is combined with Gwl inhibition (new Figure 8B-C). We have therefore revised the text to state explicitly that MARK3 should be considered a direct substrate and a putative contributor, but not the main mechanistic link between Greatwall and cytokinesis.

4. This is probably the biggest weakness. The authors demonstrate reduced proliferation and viability in cell lines and primary AML samples, but they never test whether Greatwall inhibition suppresses leukemia growth in animal models. There are no xenograft studies, leukemia burden measurements, or survival analyses. Therefore, the therapeutic relevance of targeting Greatwall in AML remains speculative.

Response: We agree with the reviewer that in vivo validation would strengthen the therapeutic relevance of targeting Greatwall. However, as this is not a translational study, animal experimentation is beyond the scope of the present investigation, which is instead centered on defining a cell-biological mechanism of mitotic regulation rather than establishing preclinical efficacy.

Instead of claiming therapeutic relevance, our data demonstrate that Greatwall inhibition induces robust cytokinetic defects and loss of viability across a broad panel of AML cell lines and primary patient samples. Crucially, these mitotic defects arise independently of the canonical ENSA–PP2A-B55 pathway, revealing a previously unappreciated mode of Greatwall function in cytokinesis. These conceptual advances challenge the textbook

view of mitotic control and establishes a mechanistic foundation upon which future in vivo studies can be justified.

5. The study lacks key genetic rescue experiments that are essential for establishing causality in the proposed pathway. Specifically, it does not include re-expression of shRNA-resistant Greatwall to confirm that the observed phenotypes are specifically due to Greatwall depletion. In addition, expression of constitutively active MARK3 would be important to determine whether downstream signaling can be restored independently of upstream inhibition. A critical missing element is also the demonstration that cytokinesis defects can be rescued upon restoration of either Greatwall or MARK3 activity. Without these rescue experiments, it remains difficult to rule out indirect or off-target effects as the underlying cause of the observed phenotypes.

Response:

We agree with the reviewer that genetic rescue experiments can be useful to establish causality. However, in our system these approaches are not technically feasible. Greatwall depletion in AML suspension cells results in a partial knockdown (~60%), and under these conditions ectopic overexpression of a shRNA-resistant Greatwall construct is unlikely to constitute a meaningful rescue. Rather than restoring endogenous levels, it would most likely introduce non-physiological overexpression. It could be argued that the appropriate control for shRNA specificity would be removal of IPTG to reverse shRNA induction. However, specificity in our studies is instead demonstrated by the fact that Gwl targeting by either a small molecule or shRNA produced the same phenotypes.

Regarding MARK3, as detailed in our response to Comment 2, MARK3 is a direct Gwl substrate, but new data shown in the revised manuscript demonstrate that it is not the main effector of the cytokinetic defects induced by Gwl inhibition. Therefore, expression of constitutively active MARK3 would not be expected to revert the phenotype. These concordant biochemical effects strongly support the specificity of the genetic perturbation.

1st Revision - Editorial Decision **9th Sep 2026**

Dear Prof. Cutillas,

Thank you for the submission of your revised manuscript to our editorial offices. I have now received the reports from three referees that were asked to re-evaluate the study, you will find below. As you will see, the referees now fully support publication of your study in EMBO reports.

Before I can proceed with formal acceptance, I have the editorial requests below I ask you to address in a final revised manuscript. Please also provide a final p-b-p-response to the editorial requests.

Editorial requests:

- There are name discrepancies: It is Tommy Shields in the manuscript vs. Tommy Shields Shields in the submission system and Pedro Casado in the manuscript vs. Pedro Casado-Izquierdo in the system. Please check and make sure the correct and same names are used.

- Please order the manuscript sections like this, using only these names:
Title page - Abstract - Keywords (max. 5) - Introduction - Results - Discussion - Methods - Data availability section - Acknowledgements (including funding information) - Disclosure and Competing Interests Statement - References - Figure legends - Expanded View Figure legends

Thus, please move the funding section to the Acknowledgements.

- We now use CRediT to specify the contributions of each author in the journal submission system. CRediT replaces the author contribution section. Please use the free text box to provide more detailed descriptions and do NOT provide your final manuscript text file with an author contributions section. See also our guide to authors (section 'Author contributions'):
https://link.springer.com/journal/44319/submission-guidelines#cms-Revised-submissions

- The Data availability section (DAS) is restricted to large datasets produced during the study and deposited. Thus, please mention here only the datasets deposited at the ProteomeXchange Consortium. Please remove now the referee access information and add final links (specific URLs) to directly reach these datasets. Finally, make sure that these datasets are public latest upon online publication of the manuscript.

- Published datasets used for the study need to be included as data citations in the reference list (and must not be part of the DAS). Data citations in the article text are distinct from normal bibliographical citations and should directly link to the database records from which the data can be accessed. In the main text, data citations are formatted as follows: "Data ref: Smith et al, 2001" or "Data ref: NCBI Sequence Read Archive PRJNA342805, 2017". In the Reference list, data citations must be labeled with "[DATASET]". A data reference must provide the database name, accession number/identifiers and a resolvable link to the landing page from which the data can be accessed at the end of the reference.

- Please check again that the number "n" for how many independent experiments were performed, their nature (biological versus technical replicates), the bars and error bars (e.g. SEM, SD) and the test used to calculate p-values is indicated in the respective figure legends (main and EV Figures). Please also check again that all the p-values are explained in the legend, and that these fit to those shown in the figure. Please provide statistical testing where applicable. Please avoid the phrase 'independent experiment' but clearly state if these were biological or technical replicates. Please also indicate (e.g. with n.s.) if testing was performed, but the differences are not significant. In case n=2, please show the data as separate datapoints without error bars and statistics. See also:

https://link.springer.com/journal/44319/submission-guidelines#cms-Figure-and-data-presentation

If n<5, please show single datapoints for diagrams. Please make sure that for all diagrams with n>2 statistical testing has been dome and that 'n.s.' is always indicated. Moreover:
- Please note that the exact p values are not provided in the legends of 1C; 4A,D; 5A; 6F; 7G,I; 8A; EV-2C,D.
- Please note that the box plots need to be defined in terms of centre, minima, maxima, and whiskers, bounds of box and percentile in the legends of figures 1C; 2D; 4A,L; 7H,I,J; 8A; EV-1C; EV-2C,D.
- Please note that information related to n is missing in the legends of figures 7H,I,J; 8A; EV-1C,D; EV-2C,D.
- Please note that the error bars are not defined in the legends of 5A-C; EV-1D.

- We do not allow 'Supplementary Methods'. Please move all methods information from the Appendix to the main manuscript Methods section and related references to the main Reference section. Then, please delete the Appendix file and remove all callouts to 'Supplementary Methods'.

- Please confirm that for all Western blot panels shown the loading control was run on the same gel as the other proteins

detected. Please note that we discourage comparisons between samples on different gels/blots, even if the samples derive from one experiment, as confounding factors reduce comparability. If unavoidable, the figure legend must state that the samples derive from the same experiment and that gels/blots were processed in parallel. If a 'representative' loading control is shown for multiple gels/blots, the intra-gel controls should be shown in the source data files, and the figure legends should describe the data displayed accurately. See our author guidelines:

https://link.springer.com/journal/44319/submission-guidelines#cms-Figure-and-data-presentation (section 'Electrophoretic gels and blots').

- Please make sure that all figure panels are called out separately and sequentially. Presently, it seems a separate callout for panel 7C is missing. Please check.

- Please remove the instructions text from the Reagents & Tools table.

In addition, I would need from you uploaded separately:
- a short, two-sentence summary of the manuscript (not more than 35 words).
- two to four short (!) bullet points highlighting the key findings of your study (two lines each).
- a schematic summary figure as separate file that provides a sketch of the major findings (not a data image) in jpeg or tiff format (with the exact width of 550 pixels and the exact height of 300 pixels) that can be used as a visual synopsis on our website. Please upload this separately and remove the graphical abstract from the manuscript text file.

I look forward to seeing the further revised version of your manuscript when it is ready. Please let me know if you have questions regarding the revision.

Best,

Achim Breiling
Senior Editor
EMBO Reports

Referee #1:

The authors have addressed my concerns by either providing a deeper explanation or new experiments. I think the paper provides important new biological insights into MASTL and I support publication.

Referee #2:

I thank the authors for their careful responses and revisions. Overall, as said before, I find the manuscript interesting and technically solid, and I appreciate the Authors' explanations regarding the limitations and scope of the study. My main reservation remains that the context-dependent effects of Gwl are not yet integrated into a unified mechanistic model and therefore remain, to some extent, a collection of interesting observations rather than a fully resolved mechanism. Nevertheless, addressing this would require substantial additional work, and I consider the Authors' responses and revisions sufficient to address my concerns within the scope of the present manuscript.

Referee #3:

The authors have addressed all of my concerns satisfactorily. I have no further questions or comments, and I have no additional concerns regarding the manuscript.

All minor editorial requests have been addressed by the authors.

Prof. Pedro Cutillas
Instituto de Parasitología y Biomedicina López-Neyra,
Consejo Superior de Investigaciones Científicas (CSIC),
Granada 18016
Spain

Dear Prof. Cutillas,

I am very pleased to accept your manuscript for publication in the next available issue of EMBO reports. Thank you for your contribution to our journal.

Your manuscript will be processed for publication by EMBO Press. It will be copy edited and you will receive page proofs prior to publication. Please note that you will be contacted by Springer Nature Author Services to complete licensing and payment information.

You may qualify for financial assistance for your publication charges - either via a Springer Nature fully open access agreement or an EMBO initiative. Check your eligibility: https://link.springer.com/journal/44319/how-to-publish-with-us

Should you be planning a Press Release on your article, please notify the production team via this form in order to coordinate publication and release dates: https://forms.cloud.microsoft/e/eH4A8JVP6g

If you have any questions, please do not hesitate to contact the Editorial Office. Thank you for your contribution to EMBO Reports.

Yours sincerely,

Achim Breiling
Senior Editor
EMBO Reports

>>> Please note that it is EMBO Reports policy for the transcript of the editorial process (containing referee reports and your response letter) to be published as an online supplement to each paper. If you do NOT want this, you will need to inform the Editorial Office via email immediately. More information is available here: https://link.springer.com/partners/embo-press/editorial-policies#Peer%20review

EMBO Press Author Checklist

Corresponding Author Name: Sandra M. Martin-Guerrero; Helfrid Hochegger; Pedro R. Cutilla	
Journal Submitted to: EMBO Reports	
Manuscript Number: EMBOR-2026-64239V2	

USEFUL LINKS FOR COMPLETING THIS FORM
The EMBO Journal - Author Guidelines
EMBO Reports - Author Guidelines
Molecular Systems Biology - Author Guidelines
EMBO Molecular Medicine - Author Guidelines

Reporting Checklist for Life Science Articles (updated January
This checklist is adapted from Materials Design Analysis Reporting (MDAR) Checklist for Authors. MDAR establishes a minimum set of requirements in transparent reporting in the life sciences (see Statement of Task: 10.31222/osf.io/9sm4x). Please follow the journal's guidelines in preparing your
Please note that a copy of this checklist will be published alongside your article.

Abridged guidelines for figures
1. Data
The data shown in figures should satisfy the following conditions:
- the data were obtained and processed according to the field's best practice and are presented to reflect the results of the experiments in an accurate and unbiased manner.
- ideally, figure panels should include only measurements that are directly comparable to each other and obtained with the same assay.
- plots include clearly labeled error bars for independent experiments and sample sizes. Unless justified, error bars should not be shown for technical
- if n<5, the individual data points from each experiment should be plotted. Any statistical test employed should be justified.
- Source Data should be included to report the data underlying figures according to the guidelines set out in the authorship guidelines on Data

2. Captions
Each figure caption should contain the following information, for each panel where they are relevant:
- a specification of the experimental system investigated (eg cell line, species name).
- the assay(s) and method(s) used to carry out the reported observations and measurements.
- an explicit mention of the biological and chemical entity(ies) that are being measured.
- an explicit mention of the biological and chemical entity(ies) that are altered/varied/perturbed in a controlled manner.
- the exact sample size (n) for each experimental group/condition, given as a number, not a range;
- a description of the sample collection allowing the reader to understand whether the samples represent technical or biological replicates (including how many animals, litters, cultures, etc.).
- a statement of how many times the experiment shown was independently replicated in the laboratory.
- definitions of statistical methods and measures:
  - common tests, such as t-test (please specify whether paired vs. unpaired), simple χ2 tests, Wilcoxon and Mann-Whitney tests, can be unambiguously identified by name only, but more complex techniques should be described in the methods section;
  - are tests one-sided or two-sided?
  - are there adjustments for multiple comparisons?
  - exact statistical test results, e.g., P values = x but not P values < x;
  - definition of 'center values' as median or average;
  - definition of error bars as s.d. or s.e.m.

Please complete ALL of the questions below.
Select "Not Applicable" only when the requested information is not relevant for your study.

Materials

Newly Created Materials	Information included in the manuscript?	In which section is the information available? (Reagents and Tools Table, Materials and Methods, Figures, Data Availability Section)
New materials and reagents need to be available; do any restrictions apply?	Not Applicable	

Antibodies	Information included in the manuscript?	In which section is the information available? (Reagents and Tools Table, Materials and Methods, Figures, Data Availability Section)
For **antibodies** provide the following information:<br>- Commercial antibodies: RRID (if possible) or supplier name, catalogue number and or/clone number<br>- Non-commercial: RRID or citation	Yes	Reagents and Tools Table, Material and Methods

DNA and RNA sequences	Information included in the manuscript?	In which section is the information available? (Reagents and Tools Table, Materials and Methods, Figures, Data Availability Section)
**Short novel DNA or RNA including primers, probes**: provide the sequences.	Not Applicable	

Cell materials	Information included in the manuscript?	In which section is the information available? (Reagents and Tools Table, Materials and Methods, Figures, Data Availability Section)
**Cell lines:** Provide species information, strain. Provide accession number in repository **OR** supplier name, catalog number, clone number, and/**OR** RRID.	Yes	Reagents and Tools Table, Material and Methods
**Primary cultures:** Provide species, strain, sex of origin, genetic modification status.	Yes	Reagents and Tools Table, Material and Methods
Report if the cell lines were recently **authenticated** (e.g., by STR profiling) and tested for mycoplasma contamination.	Yes	Material and Methods

Experimental animals	Information included in the manuscript?	In which section is the information available? (Reagents and Tools Table, Materials and Methods, Figures, Data Availability Section)
**Laboratory animals or Model organisms:** Provide species, strain, sex, age, genetic modification status. Provide accession number in repository **OR** supplier name, catalog number, clone number, **OR** RRID.	Not Applicable	
**Animal observed in or captured from the field:** Provide species, sex, and age where possible.	Not Applicable	
Please detail **housing and husbandry conditions**.	Not Applicable	

Plants and microbes	Information included in the manuscript?	In which section is the information available? (Reagents and Tools Table, Materials and Methods, Figures, Data Availability Section)
**Plants:** provide species and strain, ecotype and cultivar where relevant, unique accession number if available, and source (including location for collected wild specimens).	Not Applicable	
**Microbes:** provide species and strain, unique accession number if available, and source.	Not Applicable	

Human research participants	Information included in the manuscript?	In which section is the information available? (Reagents and Tools Table, Materials and Methods, Figures, Data Availability Section)
If collected and within the bounds of privacy constraints report on age, sex and gender or ethnicity for all study participants.	Not Applicable	

Core facilities	Information included in the manuscript?	In which section is the information available? (Reagents and Tools Table, Materials and Methods, Figures, Data Availability Section)
If your work benefited from core facilities, was their service mentioned in the acknowledgments section?	Yes	Acknowledgments section

Design

Study protocol	Information included in the manuscript?	In which section is the information available? (Reagents and Tools Table, Materials and Methods, Figures, Data Availability Section)
If study protocol has been **pre-registered, provide DOI in the manuscript**. For clinical trials, provide the trial registration number **OR** cite DOI.	Not Applicable	
Report the **clinical trial registration number** (at ClinicalTrials.gov or equivalent), where applicable.	Not Applicable	

Laboratory protocol	Information included in the manuscript?	In which section is the information available? (Reagents and Tools Table, Materials and Methods, Figures, Data Availability Section)
Provide DOI OR other citation details if **external detailed step-by-step protocols** are available.	Yes	Reagents and Tools Table, Material and Methods, Supplementary Information

Experimental study design and statistics	Information included in the manuscript?	In which section is the information available? (Reagents and Tools Table, Materials and Methods, Figures, Data Availability Section)
Include a statement about **sample size** estimate even if no statistical methods were used.	Yes	Reagents and Tools Table, Material and Methods, Supplementary Information
Were any steps taken to minimize the effects of subjective bias when allocating animals/samples to treatment (e.g. **randomization procedure**)? If yes, have they been described?	Not Applicable	
Include a statement about **blinding** even if no blinding was done.	Not Applicable	
Describe **inclusion/exclusion criteria** if samples or animals were excluded from the analysis. Were the criteria pre-established?<br><br>If sample or data points were omitted from analysis, report if this was due to attrition or intentional exclusion and provide justification.	Not Applicable	
For every figure, are **statistical tests** justified as appropriate? Do the data meet the assumptions of the tests (e.g., normal distribution)? Describe any methods used to assess it. Is there an estimate of variation within each group of data? Is the variance similar between the groups that are being statistically compared?	Yes	Figure legends, Material and Methods, Supplementary Information

Sample definition and in-laboratory replication	Information included in the manuscript?	In which section is the information available? (Reagents and Tools Table, Materials and Methods, Figures, Data Availability Section)
In the figure legends: state number of times the experiment was **replicated** in laboratory.	Yes	Figure legends, Material and Methods, Supplementary Information
In the figure legends: define whether data describe **technical or biological replicates**.	Yes	Figure legends

Ethics

Ethics	Information included in the manuscript?	In which section is the information available? (Reagents and Tools Table, Materials and Methods, Figures, Data Availability Section)
Studies involving **human participants**: State details of **authority granting ethics approval** (IRB or equivalent committee(s), provide reference number for approval.	Yes	Materials and Methods
Studies involving **human participants**: Include a statement confirming that **informed consent** was obtained from all subjects and that the experiments conformed to the principles set out in the WMA Declaration of Helsinki and the Department of Health and Human Services Belmont Report.	Yes	Materials and Methods
Studies involving **human participants:** For publication of **patient photos**, include a statement confirming that consent to publish was obtained.	Not Applicable	
Studies involving experimental **animals**: State details of **authority granting ethics approval** (IRB or equivalent committee(s), provide reference number for approval. Include a statement of compliance with ethical regulations.	Not Applicable	
Studies involving **specimen and field samples:** State if relevant **permits** obtained, provide details of authority approving study; if none were required, explain why.	Not Applicable	

Dual Use Research of Concern (DURC)	Information included in the manuscript?	In which section is the information available? (Reagents and Tools Table, Materials and Methods, Figures, Data Availability Section)
Could your study fall under dual use research restrictions? Please check biosecurity documents and list of **select agents and toxins** (CDC): https://www.selectagents.gov/sat/list.htm	Not Applicable	
If you used a select agent, is the security level of the lab appropriate and reported in the manuscript?	Not Applicable	
If a study is subject to dual use research of concern regulations, is the name of the **authority granting approval and reference number** for the regulatory approval provided in the manuscript?	Not Applicable	

Reporting

The MDAR framework recommends adoption of discipline-specific guidelines, established and endorsed through community initiatives. Journals have their own policy about requiring specific guidelines and recommendations to complement MDAR.

Adherence to community standards	Information included in the manuscript?	In which section is the information available? (Reagents and Tools Table, Materials and Methods, Figures, Data Availability Section)
State if relevant guidelines or checklists (e.g., **ICMJE, MIBBI, ARRIVE, PRISMA**) have been followed or provided.	Not Applicable	
For **tumor marker prognostic studies,** we recommend that you follow the **REMARK** reporting guidelines (see link list at top right). See author guidelines, under 'Reporting Guidelines'. Please confirm you have followed these guidelines.	Not Applicable	
For **phase II and III randomized controlled trials**, please refer to the **CONSORT** flow diagram (see link list at top right) and submit the CONSORT checklist (see link list at top right) with your submission. See author guidelines, under 'Reporting Guidelines'. Please confirm you have submitted this list.	Not Applicable	

Data Availability

Data availability	Information included in the manuscript?	In which section is the information available? (Reagents and Tools Table, Materials and Methods, Figures, Data Availability Section)
Have **primary datasets** been deposited according to the journal's guidelines (see 'Data Deposition' section) and the respective accession numbers provided in the Data Availability Section?	Yes	Data Availability Section
Were **human clinical and genomic datasets** deposited in a public access-controlled repository in accordance to ethical obligations to the patients and to the applicable consent agreement?	Not Applicable	
Are **computational models** that are central and integral to a study available without restrictions in a machine-readable form? Were the relevant accession numbers or links provided?	Not Applicable	
If publicly available data were reused, provide the respective **data citations in the reference list**.	Yes	Results section, Material and Methods and Data Availability Section
